# Extracellular CIRP activates STING to exacerbate hemorrhagic shock

**DOI:** 10.1172/jci.insight.143715

**Published:** 2021-07-22

**Authors:** Kehong Chen, Joaquin Cagliani, Monowar Aziz, Chuyi Tan, Max Brenner, Ping Wang

**Affiliations:** 1 Center for Immunology and Inflammation, The Feinstein Institutes for Medical Research, Manhasset, New York, USA.; 2 Department of Surgery and Molecular Medicine, Zucker School of Medicine at Hofstra/Northwell, Manhasset, New York, USA.

**Keywords:** Inflammation, Bacterial infections

## Abstract

Stimulator of IFN genes (STING) activates TANK-binding kinase 1 (TBK1) and IFN regulatory factor 3 (IRF3) to produce type I IFNs. Extracellular cold-inducible RNA-binding protein (eCIRP) is released from cells during hemorrhagic shock (HS). We hypothesized that eCIRP activates STING to induce inflammation and acute lung injury (ALI) after HS. WT and STING^–/–^ mice underwent controlled hemorrhage by bleeding, followed by fluid resuscitation. Blood and lungs were collected at 4 hours after resuscitation. Serum ALT, AST, LDH, IL-6, and IFN-β were significantly decreased in STING^–/–^ mice compared with WT mice after HS. In STING^–/–^ mice, the levels of pTBK1 and pIRF3, and expression of TNF-α, IL-6, and IL-1β mRNAs and proteins in the lungs, were significantly decreased compared with WT HS mice. The 10-day mortality rate in STING^–/–^ mice was significantly reduced. I.v. injection of recombinant mouse CIRP (rmCIRP) in STING^–/–^ mice showed a significant decrease in pTBK1 and pIRF3 and in IFN-α and IFN-β mRNAs and proteins in the lungs compared with rmCIRP-treated WT mice. Treatment of TLR4^–/–^, MyD88^–/–^, and TRIF^–/–^ macrophages with rmCIRP significantly decreased pTBK1 and pIRF3 levels and IFN-α and IFN-β mRNAs and proteins compared with WT macrophages. HS increases eCIRP levels, which activate STING through TLR4/MyD88/TRIF pathways to exacerbate inflammation.

## Graphical abstract

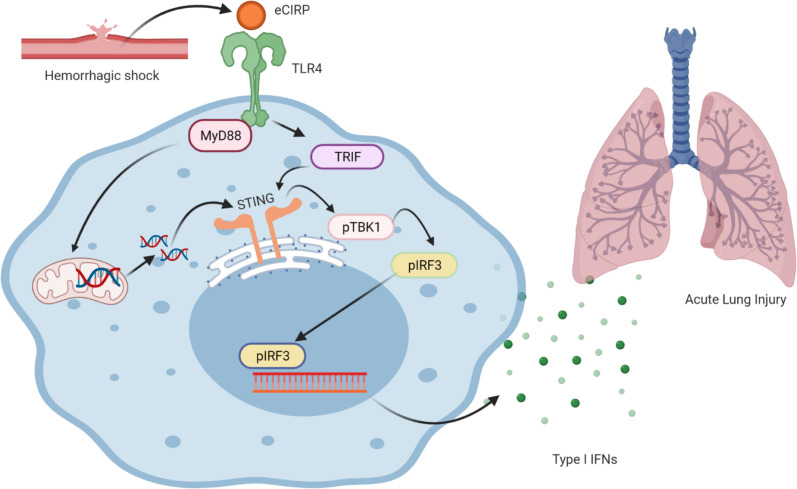

## Introduction

Loss of blood due to severe external or internal bleeding causes hemorrhagic shock (HS), responsible for nearly 40% of trauma-related deaths in the United States each year (1, 2). HS is characterized by hemodynamic instability, decreased tissue perfusion, cellular hypoxia, hyperinflammation, coagulopathy, and organ injury (3). Proinflammatory cytokines, reactive oxygen species, and nitric oxide as released from activated immune cells contribute to organ injury during and after HS (3, 4).

Cold-inducible RNA-binding protein (CIRP) is an 18-kDa RNA chaperone protein (5). During sepsis, HS, and ischemia-reperfusion (I/R) injuries, CIRP is released outside the cells through active secretion by lysosomal exocytosis and passively by cellular necrosis (6, 7). Extracellular CIRP (eCIRP) binds to its receptor TLR4 expressed in immune cells (6). TLR4 utilizes its intracytoplasmic toll/IL-1 receptor (TIR) domains for downstream signal transduction (8–10). TIR domain–containing adaptors, such as myeloid differentiation factor 88 (MyD88), Toll/IL-1 receptor domain–containing adapter protein (TIRAP), and TIR domain–containing adapter-inducing IFN-β (TRIF) play pivotal roles in TLR signaling (10, 11).

Stimulator of IFN genes (STING), also known as transmembrane protein 173 (TMEM173), detects cytosolic DNA and initiates downstream signaling for the expression of inflammatory genes (12, 13). Under normal conditions, STING is localized in the ER and is expressed mainly in hematopoietic cells, such as macrophages, DCs, NK cells, and T cells (13–15). STING is activated by cyclic dinucleotides (CDNs) secreted by intracellular bacteria or noncanonically by cyclic GMP-AMP (cGAMP) generated by cyclic GMP-AMP synthase (cGAS) (14–16). The sensing of CDNs induces a conformational change in STING and triggers the trafficking of STING complexed with TANK-binding kinase 1 (TBK1) from the ER to endosomal and lysosomal perinuclear regions (14, 15, 17). Translocated TBK1 leads to the activation of transcription factors IFN regulatory factor 3 (IRF3) and NF-κB, which translocate to the nucleus and initiate the expression of type I IFNs and other proinflammatory genes (12, 15). eCIRP stimulation leads to mitochondrial DNA fragmentation after severe trauma (18). Mitochondrial DNA fragments serve as one of the ligands that cause STING activation, resulting in the production of type I IFNs and proinflammatory cytokines (19).

In this study, we hypothesized that eCIRP activates STING pathway, causing hyperinflammation and acute lung injury (ALI) after HS. We found that the deficiency of STING resulted in decreased inflammation and ALI and improved the survival rate in HS. We also proved that eCIRP directly activated STING signaling in vitro and in vivo to produce more type I IFNs. We further identified that eCIRP activates STING through the TLR4-MyD88 pathway combined with the fragmented extramitochondrial DNA– and TRIF-dependent pathway. These findings uncover potentially novel pathobiology of eCIRP in HS and potential therapeutic avenues to protect the lungs from HS-induced ALI.

## Results

### STING^–/–^ mice are associated with decreased levels of circulating tissue injury and inflammatory markers and an increased survival rate after HS.

**Figure 1:**
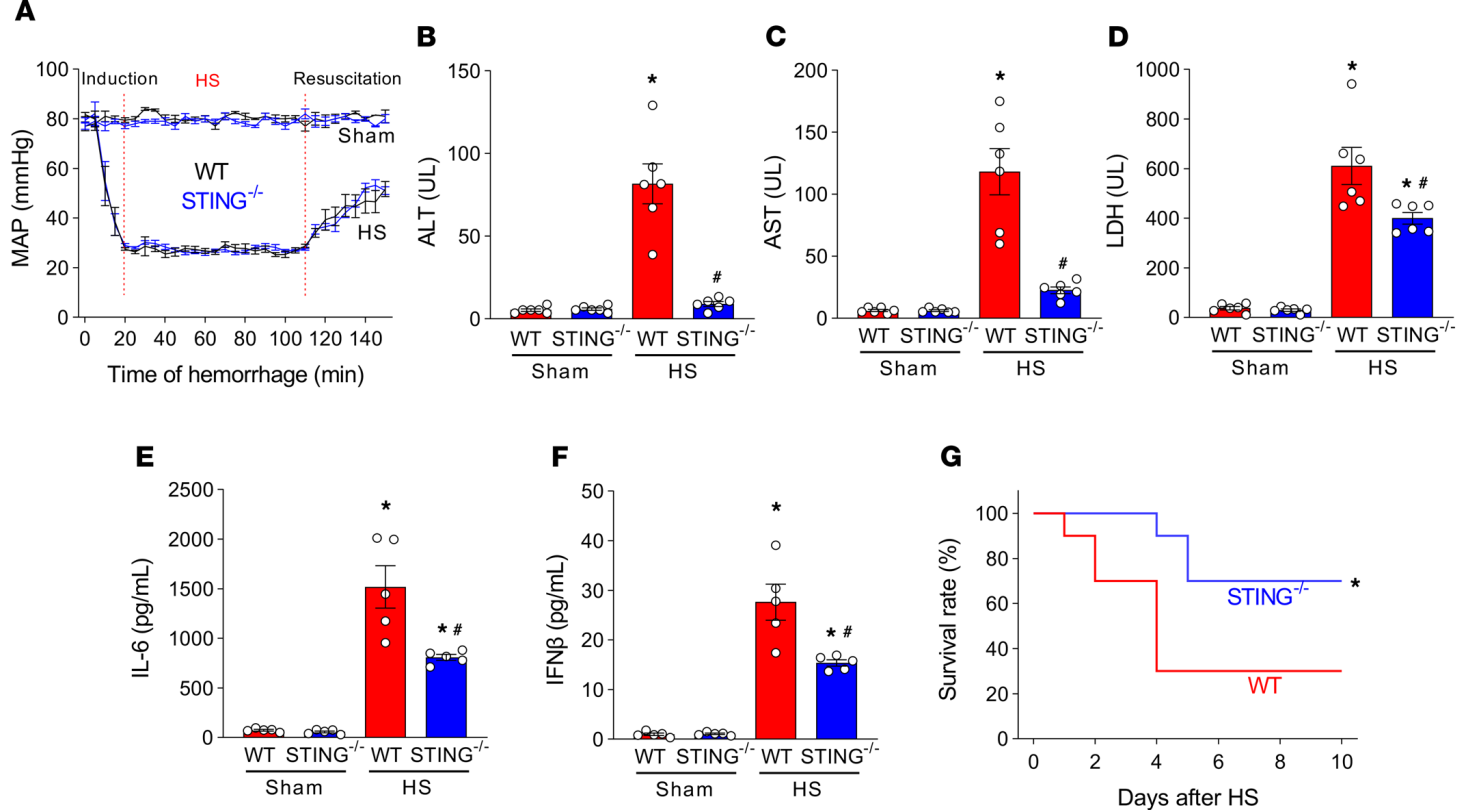
STING^–/–^ mice have less serum organ injury and inflammatory markers, and they exhibit survival benefit in HS. (**A**) HS was induced in WT and STING^–/–^ mice, and mean arterial pressure (MAP) was recorded at the induction phase (0–20 min), the hemorrhage phase (20–110 min), and the first 30 minutes of the resuscitation phase (110–140 min). Sham-operated mice with the same time frame were also recorded. Data are expressed as mean ± SEM (*n* = 6 mice/group). (**B**–**F**) Blood from WT and STING^–/–^ mice at 4 hours after HS and sham mice were collected for the analysis of the serum levels of (**B**) ALT, (**C**) AST, (**D**) LDH, (**E**) IL-6, and (**F**) IFN-β. Data are expressed as mean ± SEM (*n* = 6 mice/group) and compared by ANOVA and SNK tests. The experiments were performed 3 times, and all data were used for analysis. *P < 0.05 versus WT-sham; ^#^*P* < 0.05 versus WT-HS mice. (**G**) HS was induced in WT and STING^–/–^ mice. Mice were monitored for survival for 10 days. Survival rates were analyzed by the Kaplan-Meier estimator using a log-rank test (*n* = 10 mice/group; *P < 0.05 versus WT-HS). HS, hemorrhagic shock; ALT, alanine aminotransferase; AST, aspartate aminotransferase; LDH, lactate dehydrogenase.

We recorded mean arterial pressure (MAP) during 90 minutes of controlled hemorrhage and 30 minutes of fluid resuscitation in WT and STING^–/–^ mice. In both groups of animals during hemorrhage, there was a sharp decline in MAP, and fluid resuscitation partially restored MAP in a similar fashion (Figure 1A). To determine the impact of STING on organ injury and inflammation in HS, we assessed tissue injury markers in the serum and found that ALT, AST, and LDH levels were significantly increased in the WT mice by 15-, 19-, and 16-fold, respectively, compared with WT sham mice (Figure 1, B–D). In contrast, there were significant reductions in the serum levels of ALT, AST, and LDH by 89%, 81%, and 35%, respectively, in the STING^–/–^ mice (Figure 1, B–D). Serum IL-6 and IFN-β levels were increased by 20- and 28-fold, respectively, in WT HS mice, but they were reduced by 47% and 44% in the STING^–/–^ HS group, respectively (Figure 1, E and F). To assess whether the deficiency of STING affects mouse survival after HS, we performed a 10-day survival curve in WT and STING^–/–^ mice. The survival rate of STING^–/–^ mice was significantly higher than the WT mice (70% versus 30%) after HS (Figure 1G). Taken together, the deficiency of STING attenuates tissue injury and inflammation and reduces mortality after HS.

### STING^–/–^ mice are protected against ALI caused by HS.

**Figure 2:**
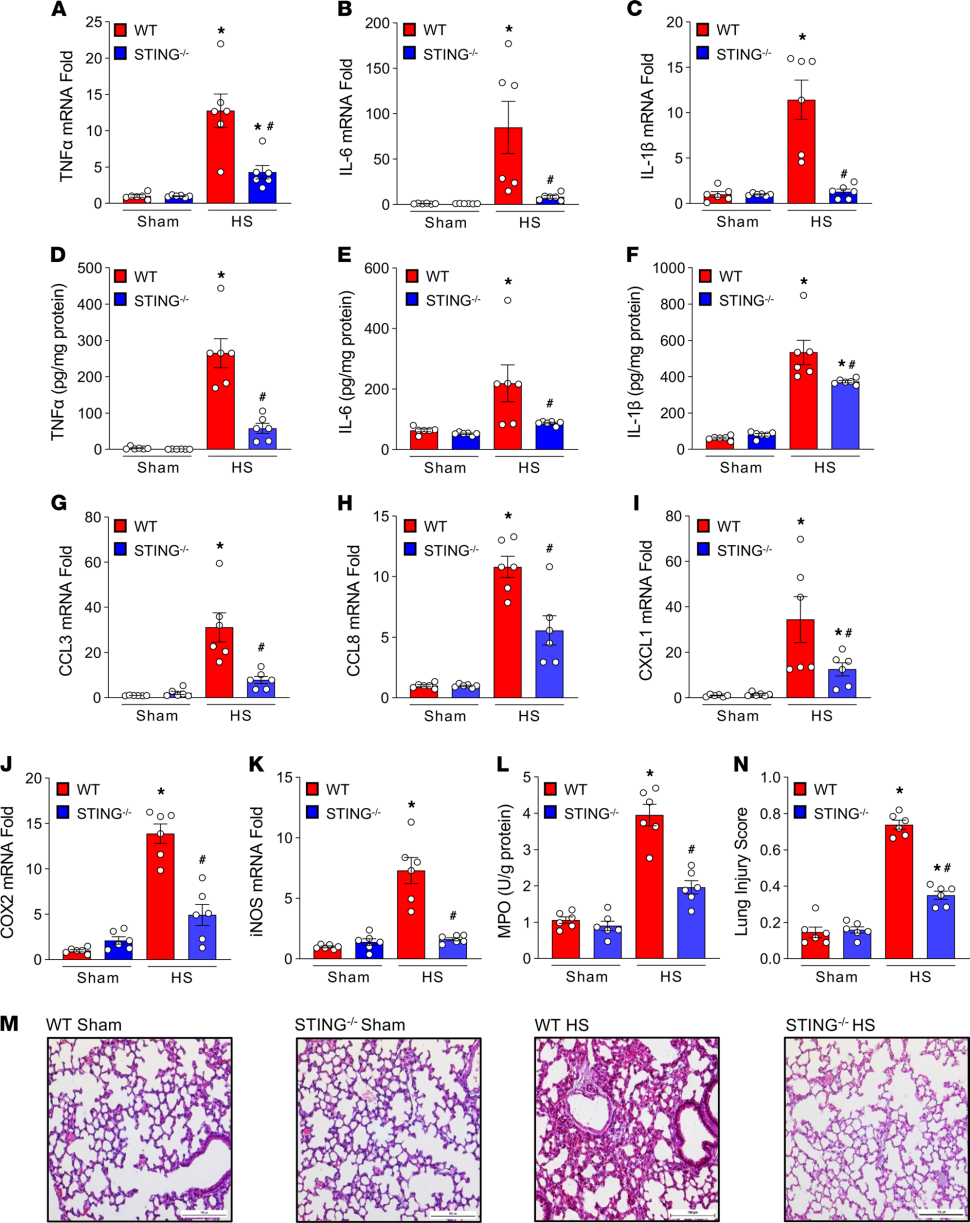
STING^–/–^ mice are protected against ALI in HS. WT and STING^–/–^ mice were subjected to sham or HS operation. After 4 hours of HS operation, lung tissues were collected from sham and HS mice. (**A**–**F**) Expression of (**A** and **D**) TNF-α, (**B** and **E**) IL-6, and (**C** and **F**) IL-1β mRNA and protein levels in lungs were assessed by real-time PCR and ELISA, respectively. (**G**–**K**) Lung tissues of WT and STING^–/–^ HS mice were assessed for the expression of (**G**) CCL3, (**H**) CCL8, (**I**) CXCL1, (**J**) COX2, and (**K**) iNOS mRNA by real-time PCR. (**L**) MPO activity in lung tissues of WT and STING^–/–^ sham and HS mice were assessed by enzymatic assay. Data are expressed as mean ± SEM. *n* = 6 mice/group. The groups were compared by 1-way ANOVA and SNK method (*P < 0.05 versus WT-sham; ^#^*P* < 0.05 versus WT-HS mice). (**M** and **N**) HS was induced in WT and STING^–/–^ mice. After 4 hours of HS, lung tissues were collected for histological analysis. (**M**) Representative images of H&E-stained lung tissue at 200×. Scale bar: 50 μm. (**N**) Lung injury scores ranged from 0 to 1 and were based on the presence of proteinaceous debris in the airspaces, the degree of septal thickening, and neutrophil infiltration in the alveolar and interstitial spaces. *n* = 6 high-power field images/group obtained from 6 mice/group. The experiments were performed 2 times, and all data were used for analysis. Data are expressed as mean ± SEM and compared by 1-way ANOVA and SNK method (*P < 0.05 versus WT-sham; ^#^*P* < 0.05 versus WT-HS mice).

We assessed the expression of proinflammatory cytokines in the lungs of WT and STING^–/–^ mice after HS. Our previous study demonstrates that 4 hours after HS is sufficient to produce significantly higher levels of cytokines (6); we chose the 4-hour time point to study proinflammatory cytokines in HS mice. In WT mice, the mRNAs of TNF-α, IL-6, and IL-1β in lungs were significantly increased by 13-, 85-, and 11-fold, respectively, in HS, compared with sham mice (Figure 2, A–C). However, in STING^–/–^ HS mice, the expression of TNF-α, IL-6, and IL-1β mRNAs in the lungs was significantly decreased by 66%, 90%, and 89%, respectively, compared with WT HS mice (Figure 2, A–C). Similarly, protein levels of TNF-α, IL-6, and IL-1β in lungs was significantly increased in WT HS mice. In contrast, in the STING^–/–^ HS mice, the expression of these cytokines at protein levels were significantly decreased by 78%, 60%, and 30%, respectively (Figure 2, D–F). The expression of CCL3, CCL8, and CXCL1 at mRNA levels in lungs was increased in WT mice after HS (Figure 2, G–I). However, their expression was significantly reduced by 75%, 48%, and 63%, respectively, in STING^–/–^ HS mice (Figure 2, G–I). In WT HS mice, the expression of COX2 and iNOS mRNAs was increased, and it was decreased by 64% and 78% in STING^–/–^ HS mice, respectively (Figure 2, J and K). The activity of granulocyte infiltration/activation marker, MPO, was increased by 4-fold in the lungs of WT mice after HS. In contrast, the MPO activity was significantly decreased by 50% in STING^–/–^ HS mice (Figure 2L). We next evaluated the degree of ALI by observing lung histology specimens in WT and STING^–/–^ mice after HS. Lung injury score showed a 5-fold increase in the WT HS mice compared with the WT sham mice, while the lung injury score was significantly decreased by 57% in STING^–/–^ HS mice (Figure 2, M and N). These data suggest that the absence of STING significantly reduces inflammation and injury of lungs by decreasing the expression of proinflammatory cytokines and chemokines, decreasing MPO activity, and improving lung histology score after HS.

### STING^–/–^ mice show the decreased activation of TBK1 and IRF3 after HS and in vivo rmCIRP injection.

**Figure 3:**
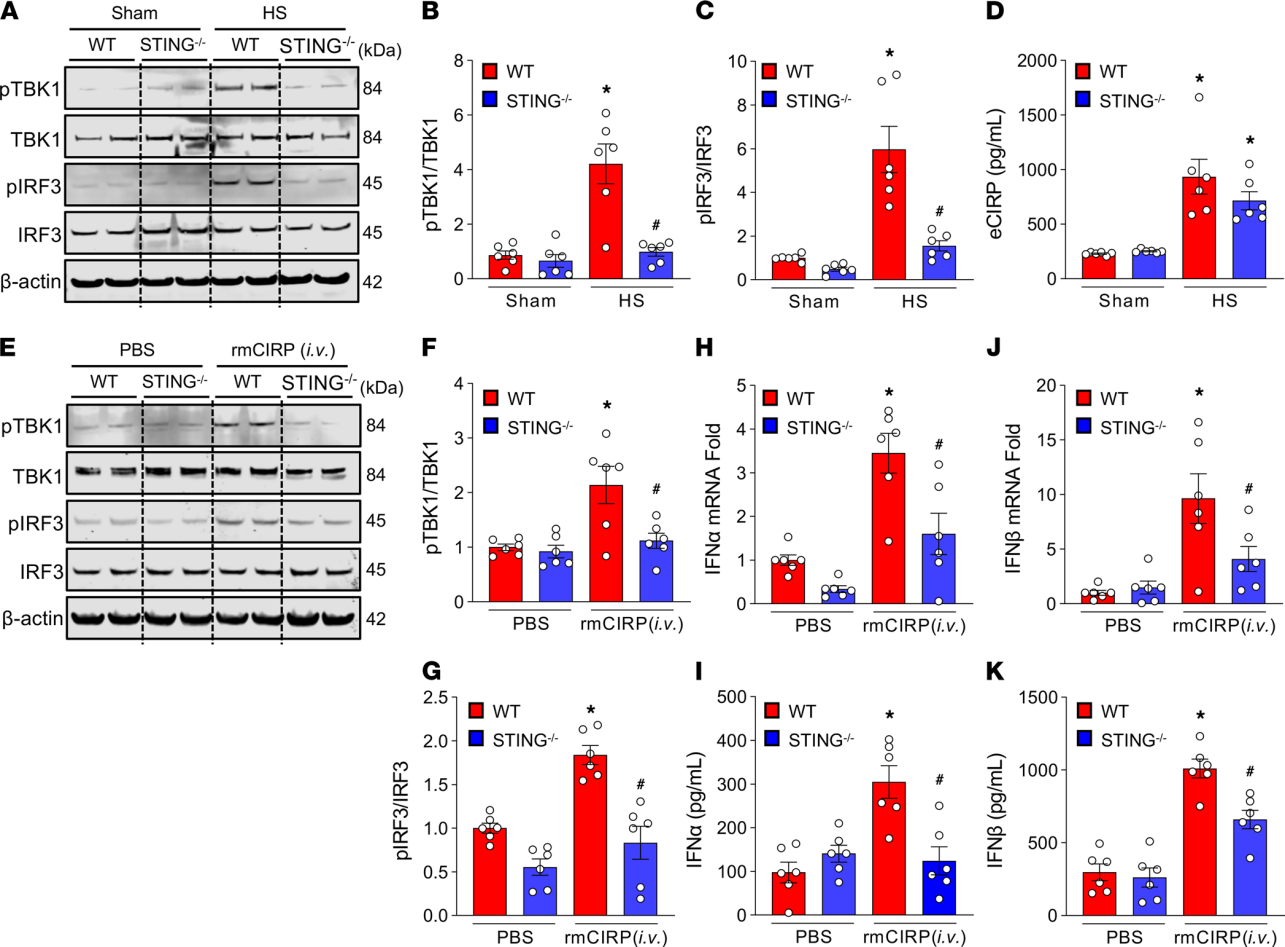
TBK1 and IRF3 in lungs in HS and rmCIRP-treated mice. (**A**–**C**) After 4 hours of HS, lungs were harvested and assessed for (**A** and **B**) pTBK1 and TBK1 and (**A** and **C**) pIRF3 and IRF3. Representative Western blots are shown. pTBK1 and pIRF3 expression in each sample was normalized to total TBK1 and IRF3 expression, and the mean values of the WT-sham group were standardized as 1 for comparison. Data are expressed as mean ± SEM (*n* = 6 mice/group) and compared by ANOVA and SNK tests (*P < 0.05 versus WT-sham; ^#^*P* < 0.05 versus WT-HS mice). (**D**) eCIRP levels in serum of WT and STING^–/–^ mice following sham or 4 hours after HS were assessed by ELISA. Data are expressed as mean ± SEM (*n* = 6 mice/group) and compared by ANOVA and SNK tests (*P < 0.05 versus WT-sham). (**E**–**G**) After 4 hours of injection with rmCIRP (5 mg/kg body weight) or equivalent volume PBS (vehicle), lungs were harvested from each group of mice and assessed for (**E** and **F**) pTBK1 and TBK1 and (**E** and **G**) pIRF3 and IRF3 proteins. Representative Western blots are shown. pTBK1 and pIRF3 expression in each sample was normalized to total TBK1 and IRF3 expression, and the mean values of the WT PBS-treated group were standardized as 1 for comparison. (**H**–**K**) Lung tissues of PBS or rmCIRP-injected WT and STING^–/–^ mice were analyzed for the expression of (**H** and **I**) IFN-α and (**J** and **K**) IFN-β at mRNA and protein levels by real-time PCR and ELISA, respectively. Data are expressed as mean ± SEM (*n* = 6 mice/group) and compared by ANOVA and SNK tests (*P < 0.05 versus PBS-injected and ^#^*P* < 0.05 versus rmCIRP-injected mice). The experiments were performed 3 times, and all data were used for analysis.

To determine whether HS directly activates the STING signaling pathway, we assessed the phosphorylation of TBK1 and IRF3 in the lungs. The results showed that phosphorylated TBK1 (pTBK1) and pIRF3 proteins were significantly increased in the lungs of WT HS mice compared with WT sham mice, while their levels were significantly reduced by 77%, and 74%, respectively, in STING^–/–^ HS mouse lungs, indicating that HS leads to the activation of the STING signaling pathway (Figure 3, A–C). We then assessed serum levels of eCIRP in HS mice and found a 4-fold increase compared with WT sham mice (Figure 3D). In STING^–/–^ mice following HS, however, eCIRP levels in the serum were found to be only 2.8-fold higher compared with STING^–/–^ sham mice (Figure 3D). The decreased release of CIRP in STING^–/–^ mice could be due to their less severe inflammation after HS. To confirm whether eCIRP promotes the activation of the STING pathway directly, we injected recombinant mouse CIRP (rmCIRP) into WT and STING^–/–^ mice i.v. We found that pTBK1 and pIRF3 levels were significantly increased in the lungs of WT mouse after i.v. rmCIRP injection. By contrast, the pTBK1 and pIRF3 levels in the lungs were significantly reduced by 48% and 55%, respectively, in STING^–/–^ mice compared with WT mice after rmCIRP (Figure 3, E–G). Following i.v. injection of rmCIRP in WT mice, the mRNA and protein levels of IFN-α and IFN-β in lungs were significantly increased compared with vehicle (PBS) mice (Figure 3, H–K). Interestingly, we found that the levels of IFN-α mRNAs and proteins in the lung tissues of rmCIRP-injected STING^–/–^ mice were significantly decreased by 54% and 59%, respectively (Figure 3, H and I), and IFN-β mRNAs and proteins in the lung tissues of rmCIRP-injected STING^–/–^ mice were decreased by 58% and 34%, respectively, compared with rmCIRP-injected WT mice (Figure 3, J and K). Similar to these findings of i.v. injection of rmCIRP, we also found that i.t. injection of rmCIRP in WT mice significantly increased the activation of pTBK1 and pIRF3 and expression of IFN-α and IFN-β mRNAs and proteins compared with vehicle mouse lungs (Supplemental Figure 1; supplemental material available online with this article; https://doi.org/10.1172/jci.insight.143715DS1). However, expression of these STING pathway markers was significantly reduced in rmCIRP-injected STING^–/–^ mice compared with WT mouse lungs (Supplemental Figure 1). These data clearly show that in vivo treatment with rmCIRP, as well as HS, directly activates STING pathway in mice.

### eCIRP induces STING pathway activation to produce type I IFNs in vitro.

**Figure 4:**
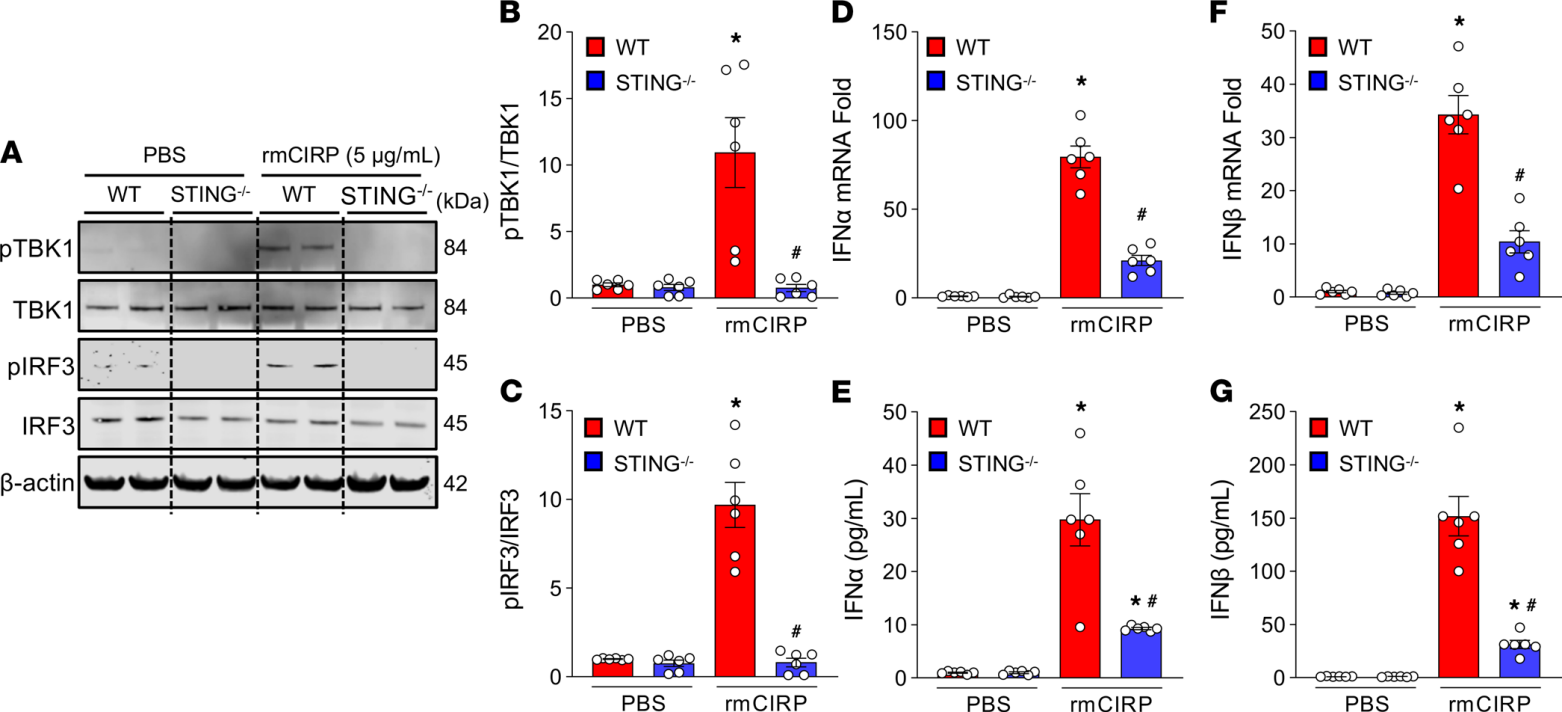
Stimulation of macrophages with rmCIRP induces type I IFN expression via STING-TBK1-IRF3 pathway. Peritoneal macrophages were isolated from WT and STING^–/–^ mice 4 days after i.p. injection of a single dose of 4% thioglycolate. A total of 2 × 10^6^ cells/mL were stimulated with 5 μg/mL of rmCIRP or an equal volume of PBS as vehicle control. (**A**–**C**) After 4 hours of stimulation with rmCIRP, total protein was extracted from each sample and assessed for (**A** and **B**) pTBK1 and TBK1 and (**A** and **C**) pIRF3 and IRF3 proteins by Western blot. The blot was stripped and incubated with anti–β-actin Abs to serve as the loading control. Representative Western blots for pTBK1, TBK1, pIRF3, IRF3, and β-actin are shown. Each blot was quantified by densitometry analysis. pTBK1 and pIRF3 expression in each sample was normalized to total TBK1 and IRF3 expression, respectively, and the mean values of the PBS-treated group were standardized as 1 for comparison. (**D**–**G**) Assessment of type I IFNs. After treatment of a total of 2 × 10^6^ cells/mL with 5 μg/mL of rmCIRP or an equal volume of PBS for 4 hours, mRNA and protein were extracted from each sample and assessed for (**D** and **E**) IFN-α and (**F** and **G**) IFN-β at mRNA and protein levels by real-time PCR and ELISA, respectively. Data are expressed as mean ± SEM (*n* = 6 samples/group) and compared by ANOVA and SNK tests (*P < 0.05 versus PBS-treated and ^#^*P* < 0.05 versus rmCIRP-treated macrophages). The experiments were performed 3 times, and all data were used for analysis.

Given that macrophages play important roles in hemorrhage-induced inflammation, we determined the activation of TBK1 and IRF3 and expression of IFN-α and IFN-β in rmCIRP-treated macrophages isolated from WT and STING^–/–^ mice. We found that rmCIRP stimulation of macrophages isolated from WT mice significantly increased the levels of pTBK1 and pIRF3 compared with vehicle-treated WT macrophages (Figure 4, A–C). However, the levels of pTBK1 and pIRF3 were significantly decreased in rmCIRP-treated STING^–/–^ mouse macrophages compared with WT macrophages by 93% and 92%, respectively (Figure 4, A–C). The expression of IFN-α and IFN-β at mRNA and protein levels were increased in rmCIRP-treated WT macrophages (Figure 4, D–G), while their expression was significantly decreased by 73% and 70%, respectively at mRNA and 69% and 79%, respectively at protein levels in rmCIRP-stimulated STING^–/–^ macrophages, compared with WT macrophages (Figure 4, D–G). These results demonstrate that eCIRP promotes STING activation to produce type I IFNs in vitro.

### eCIRP activates STING pathway through TLR4-MyD88 signaling in combination with fragmented extramitochondrial DNA.

**Figure 5:**
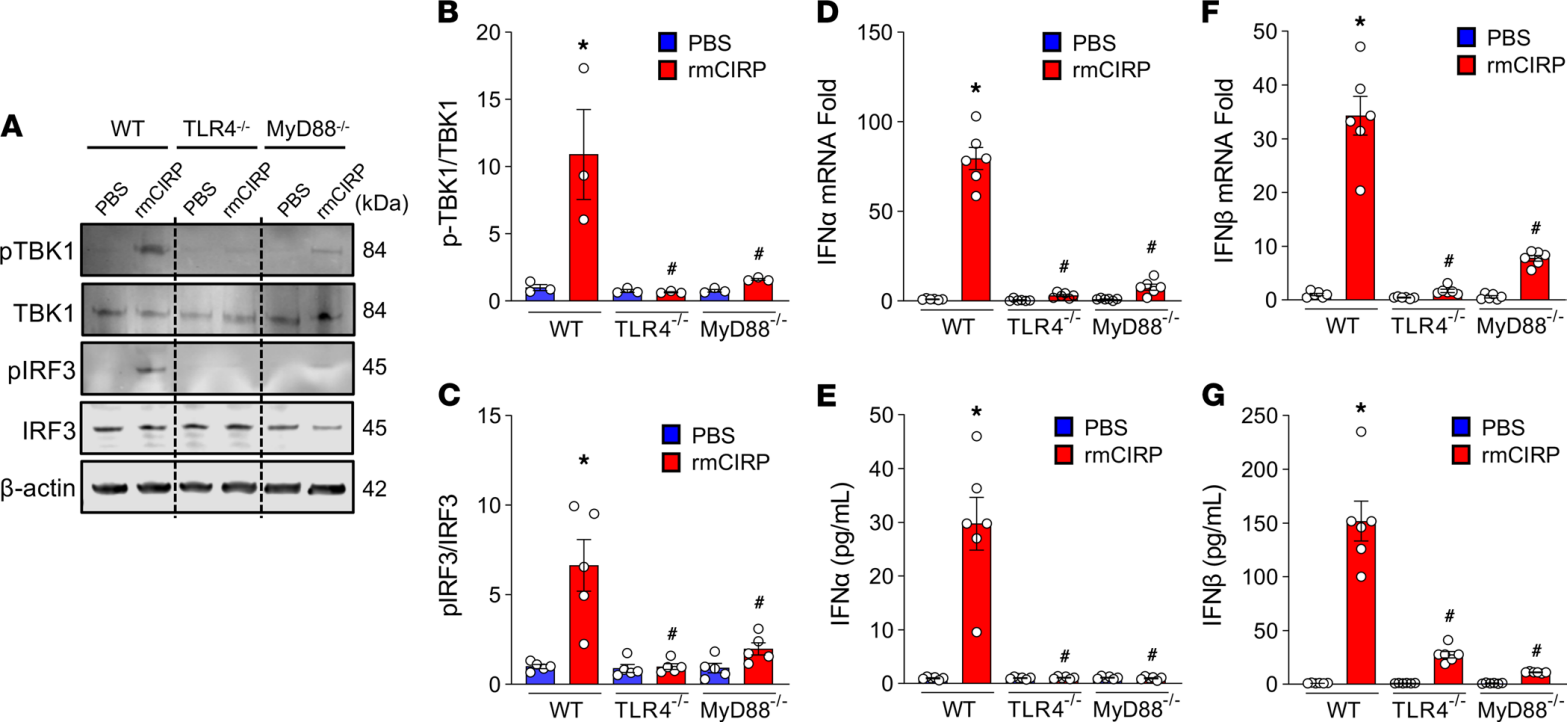
eCIRP activates STING downstream molecules through the TLR4-MyD88 pathway. Peritoneal macrophages were isolated from WT, TLR4^–/–^, and MyD88^–/–^ mice 4 days after i.p. injection of a single dose of 4% thioglycolate. A total of 2 × 10^6^ cells/mL were stimulated with 5 μg/mL rmCIRP or an equal volume of PBS as vehicle. (**A**–**C**) After 4 hours of stimulation with rmCIRP, total protein was extracted from each sample and assessed for (**A** and **B**) pTBK1 and TBK1 and (**A** and **C**) pIRF3 and IRF3 proteins by Western blot. The blot was stripped and incubated with anti-β-actin Abs to serve as the loading control. Representative Western blots for pTBK1, TBK1, pIRF3, IRF3, and β-actin are shown. Each blot was quantified by densitometry analysis. pTBK1 and pIRF3 expression in each sample was normalized to total TBK1 and IRF3 expression, respectively, and the mean values of PBS-treated WT macrophages were standardized as 1 for comparison. (**D**–**G**) Assessment of type I IFNs. Peritoneal macrophages were isolated from WT, TLR4^–/–^, and MyD88^–/–^ mice injected with thioglycolate. After treatment of a total of 2 × 10^6^ cells/mL with 5 μg/mL of rmCIRP or an equal volume of PBS for 4 hours, mRNA and protein were extracted from each sample and assessed for (**D** and **E**) IFN-α and (**F** and **G**) IFN-β at mRNA and protein levels by real-time PCR and ELISA, respectively. Data are expressed as mean ± SEM (*n* = 6 samples/group) and compared by ANOVA and SNK tests (*P < 0.05 versus PBS-treated WT macrophages; ^#^*P* < 0.05 versus rmCIRP-treated WT macrophages). The experiments were performed 3 times, and all data were used for analysis.

**Figure 6:**
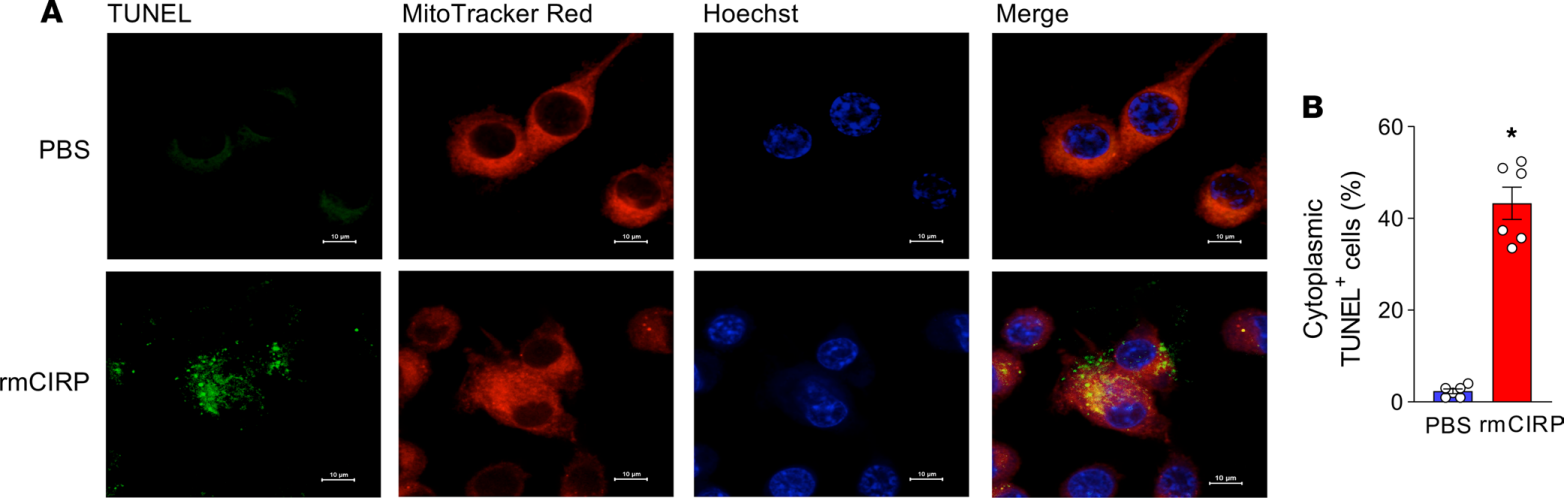
eCIRP induces mitochondrial DNA fragmentation in macrophages. After rmCIRP (1 μg/mL) stimulation of peritoneal macrophages for 4 hours, the cell culture media was discarded. MitoTracker Red was added to the culture medium. After 1 hour of culture, the cells were fixed with 2% paraformaldehyde solution for 20 minutes and then washed with PBS and blocked with normal 5% BSA for 1 hour. The cells were stained with TUNEL. The cell nucleus was stained with Hoechst 33258. The macrophages were then examined by confocal microscopy. (**A**) Representative images of the macrophages stained with TUNEL (green fluorescence), MitoTracker Red (red fluorescence), and nuclear counterstaining with Hoechst (blue fluorescence). Scale bar: 10 μM. Total original magnification, 400×. (**B**) Cells were counted in 3 random fields in each independent experiment. Data are expressed as mean ± SEM (*n* = 6 samples/group) and compared by Student’s *t* test (*P < 0.05 versus PBS-treated macrophages). The data passed the normality test (Shapiro-Wilk), with a subsequent 2-tailed *P* < 0.05. The experiments were performed 2 times, and all data were used for analysis.

WT macrophages stimulated with rmCIRP significantly increased the levels of pTBK1 and pIRF3 proteins. In contrast, the activation of these proteins was significantly decreased in rmCIRP-treated TLR4^–/–^ macrophages by 94% and 85%, respectively, and MyD88^–/–^ macrophages by 85% and 70%, respectively, compared with rmCIRP-treated WT macrophages (Figure 5, A–C). The expression of IFN-α and IFN-β at mRNA and protein was significantly increased in rmCIRP-treated WT macrophages compared with PBS-treated control. On the other hand, the expression of IFN-α and IFN-β at mRNAs and proteins were significantly decreased by 96% and 85%, respectively, at mRNA and 96% and 828%, respectively, at protein levels in rmCIRP-stimulated TLR4^–/–^ macrophages — and by 90% and 77%, respectively, at mRNA and 97% and 93%, respectively, at protein levels in rmCIRP-stimulated MyD88^–/–^ macrophages compared with WT macrophages (Figure 5, D–G). We further detected DNA fragmentation in rmCIRP-treated macrophages. We found that treatment with rmCIRP induced DNA fragmentation, and the fragmented DNA was localized with mitochondria and areas surrounding mitochondria, suggesting that rmCIRP stimulation caused the mitochondrial DNA to be fragmented, and they were released into the cytoplasm (Figure 6, A and B). These data suggest that eCIRP activates STING through the TLR4-MyD88 pathway in combination with fragmented extramitochondrial DNA.

### eCIRP induces the activation of the STING pathway through TRIF.

**Figure 7:**
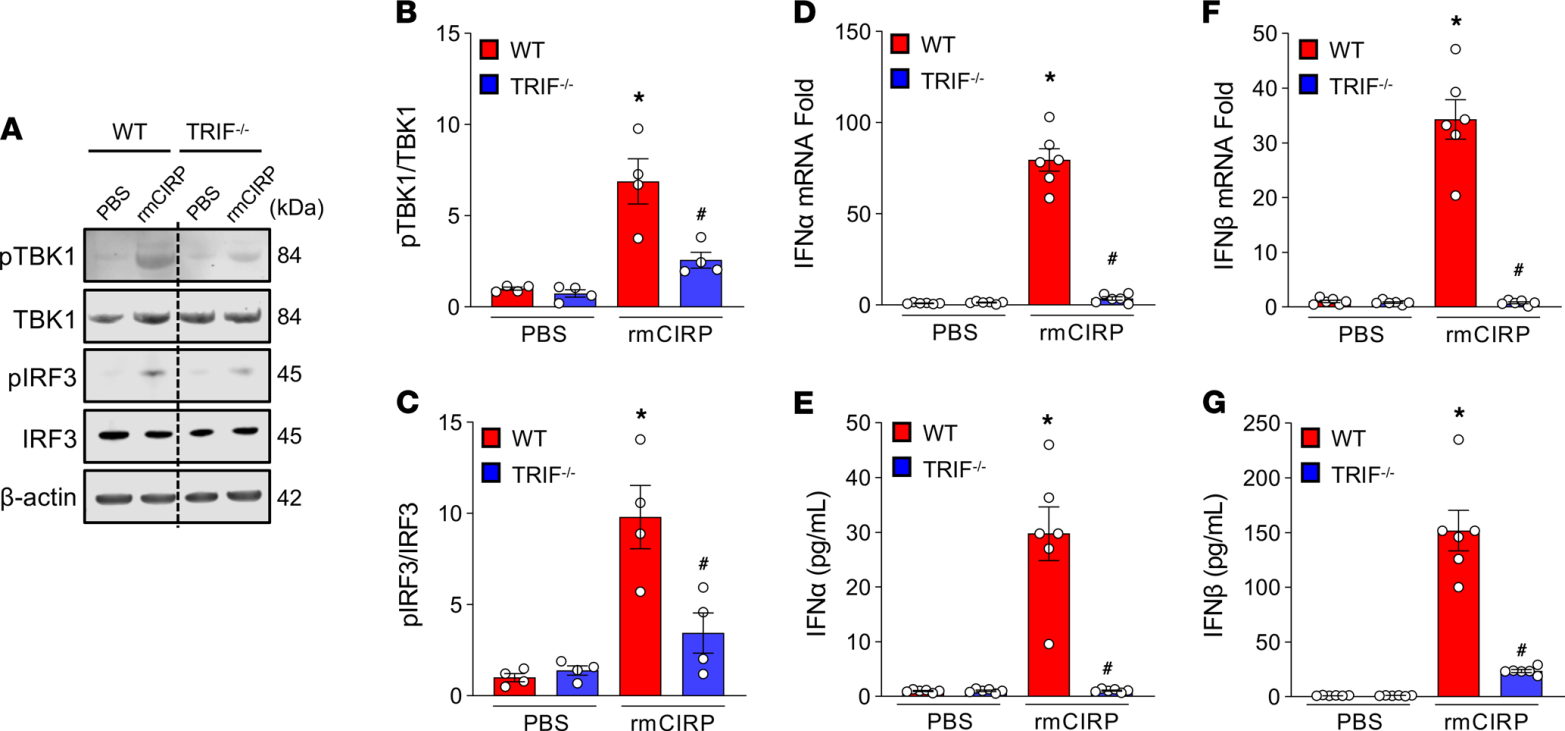
eCIRP activates the STING pathway through the TRIF-dependent pathway. Peritoneal macrophages were isolated from WT and TRIF^–/–^ mice 4 days after i.p. injection of a single dose of 4% thioglycolate. A total of 2 × 10^6^ cells/mL were stimulated with 5 μg/mL of rmCIRP or an equal volume of PBS as a vehicle. (**A**–**C**) After 4 hours of stimulation with rmCIRP, total protein was extracted from each sample and assessed for (**A** and **B**) pTBK1 and TBK1 and (**A** and **C**) pIRF3 and IRF3 proteins by Western blot. The blot was stripped and incubated with anti–β-actin Abs to serve as a loading control. Representative Western blots for pTBK1, TBK1, pIRF3, IRF3, and β-actin are shown. Each blot was quantified by densitometry analysis. pTBK1 and pIRF3 expression in each sample was normalized to total TBK1 and IRF3 expression, respectively, and the mean values of PBS-treated WT macrophages were standardized as 1 for comparison. (**D**–**G**) Assessment of type I IFNs. Peritoneal macrophages were isolated from WT and TRIF^–/–^ mice. After treatment of a total of 2 × 10^6^ cells/mL with 5 μg/mL of rmCIRP or an equal volume of PBS for 4 hours, mRNA and protein were extracted from each sample and assessed for (**D** and **E**) IFN-α and (**F** and **G**) IFN-β at mRNA and protein levels by real-time PCR and ELISA, respectively. Data are expressed as mean ± SEM (*n* = 6 samples/group) and compared by ANOVA and SNK tests (*P < 0.05 versus PBS-treated WT macrophages; ^#^*P* < 0.05 versus rmCIRP-treated WT macrophages). The experiments were performed 3 times, and all data were used for analysis.

TRIF is implicated in the TLR4- and TLR3-mediated MyD88-independent pathway to activate the immune system (20, 21). Activation of the STING pathway requires TRIF (15, 22). We, therefore, assessed the rmCIRP-induced STING pathway’s downstream molecules in TRIF^–/–^ macrophages. We found that stimulation of WT mouse macrophages with rmCIRP significantly increased the contents of pTBK1 and pIRF3 compared with PBS-treated cells (Figure 7, A–C). However, in TRIF^–/–^ macrophages, following rmCIRP treatment, the activation of TBK1 and IRF3 was significantly decreased by 62.6% and 65%, respectively, compared with rmCIRP-treated WT macrophages (Figure 7, A–C). We assessed the expression of IFN-α and IFN-β mRNA and protein expression in WT and TRIF^–/–^ macrophages. We found that, in rmCIRP-treated WT macrophages, the expression of IFN-α and IFN-β mRNA and proteins were increased. At the same time, their levels were significantly decreased in TRIF^–/–^ mouse macrophages following treatment with rmCIRP by 96% and 98% for mRNA, respectively, and 96% and 84% for protein, respectively, compared with WT macrophages (Figure 7, D–G). These results indicate that TRIF is required for eCIRP to activate STING in vitro.

## Discussion

**Figure 8:**
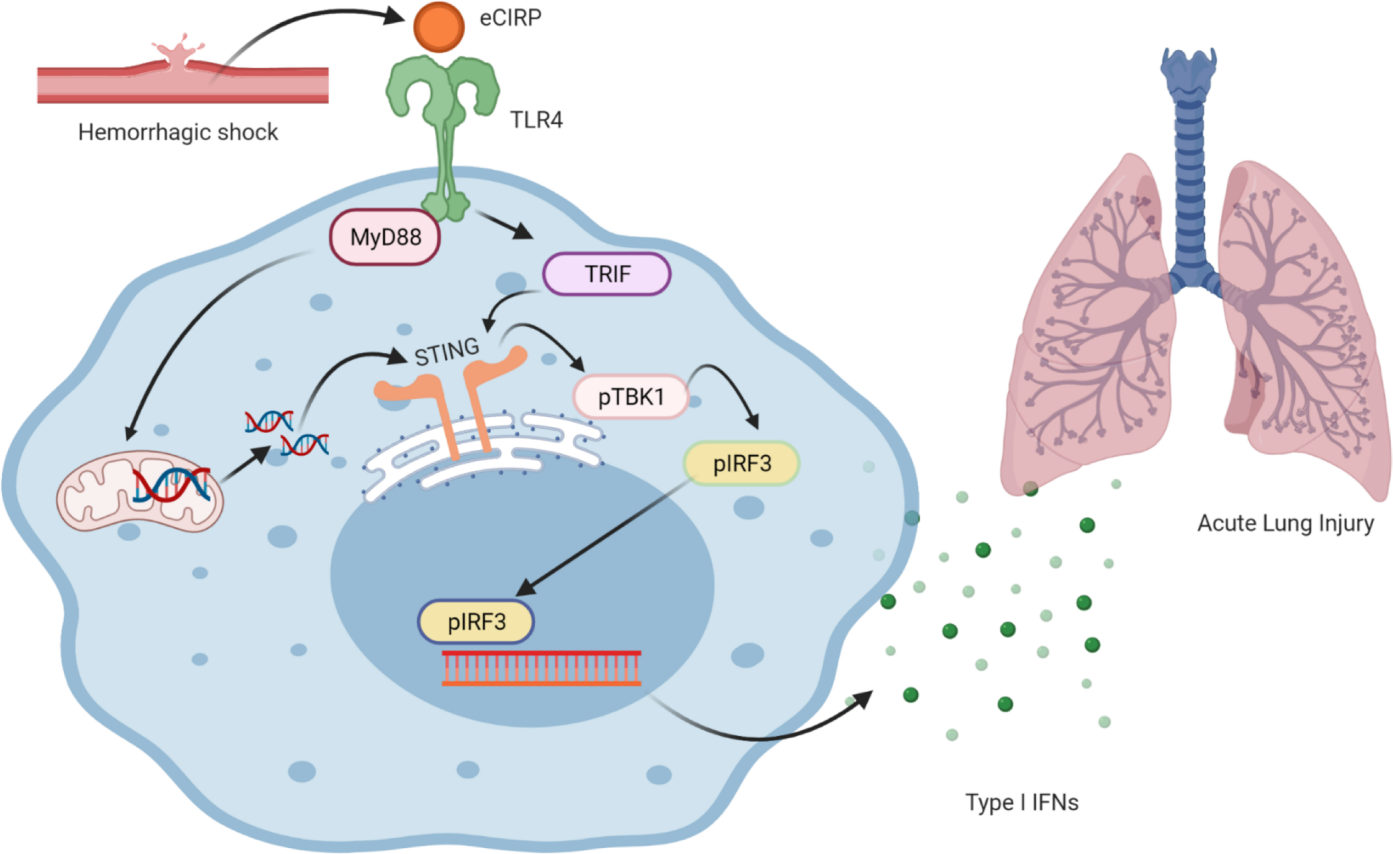
eCIRP activates the STING pathway to induce the expression of type I IFNs in hemorrhagic shock to cause acute lung injury. During HS, eCIRP is released, and it recognizes TLR4 as its receptor. TLR4 through the MyD88-dependent or -independent pathway using TRIF activates STING and its downstream molecules TBK1 and IRF3. eCIRP through the TLR4-MyD88 pathway also induces mitochondrial DNA degradation and release into the cytoplasm, which activates TBK1 through STING. IRF3 serves as a transcription factor and induces the expression of IFN-α and IFN-β to induce inflammation and tissue damage in HS. As such, HS increases eCIRP to induce the expression of type I IFNs through STING. Type I IFNs may cause further tissue injury and ALI.

eCIRP levels were significantly increased in sterile and nonsterile inflammation (6, 7, 23). In the present study, we found a significant increase in the serum levels of eCIRP in mice after HS, which established the rationale of eCIRP’s impact on the STING pathway for inducing inflammation and tissue damage in HS. TLR4 is the receptor of eCIRP (6); therefore, we primarily focused on the TLR4 pathway for eCIRP-mediated activation of STING. We revealed that the deficiency of the TLR4 and its intracellular adaptors (i.e., MyD88 and TRIF) results in decreased activation of STING’s downstream mediators, TBK1 and IRF3, and the expression of type I IFNs in HS. We also determined that eCIRP promotes mtDNA degradation and their release into the cytoplasm. A recent study showed that eCIRP promotes mitochondrial DNA damage and degradation through the TLR4-MyD88 pathway. Therefore, there is an association between TLR4-MyD88 pathway and mitochondrial DNA damage, which promotes STING activation because cytosolic DNA has been shown to induce cGAS-STING activation (12–14). Studies also showed that TRIF activation leads to STING activation (22). Based on our data and the scientific evidence, we summarized the schema as the following: eCIRP activates STING through the TLR4-MyD88 pathway, combined with the fragmented extramitochondrial DNA– and TRIF-dependent pathway (Figure 8).

Retinoic acid–inducible gene I (RIG-I) and melanoma differentiation–associated protein 5 (MDA5) are the sensors of the viral double-stranded RNA (dsRNA) to induce expression of type I IFNs during the viral infection (24, 25). By contrast, cGAS-STING serves as a pivotal sensor of cytosolic dsDNA (13). Although distinct receptors are involved in RNA and DNA sensing, the downstream signaling components are functionally interconnected, where STING serves as a critical player in this crosstalk. STING deficiency in cells and mice significantly increased the sensitivity to RNA virus infection while reducing type I IFN production (12, 13, 26). In human embryonic stem cells, STING deficiency correlated with the inability of RIG-I to produce IFN in response to cytoplasmic dsRNA (27). Nonetheless, our current study used the HS model, a sterile inflammation model in mice characterized by excessive blood loss, hypovolemic shock, tissue hypoxia, and subsequent release of DAMPs to induce inflammation. Considering RIG-I, MDA5, and mitochondrial antiviral signaling (MAVS) pathways to sense viral RNAs, our current study does not involve any viral infection model. Therefore, the involvement of RIG-I, MDA5, and MAVS pathways for STING activation may be unlikely. However, given the possible crosstalk between RIG-I–MDA5–MAVS and cGAS-STING pathways, we assessed the expression of RIG-I, MDA5, and MAVS in mouse peritoneal macrophages stimulated with rmCIRP. We found a significant increase in the expression MDA5, but not RIG-I or MAVS (Supplemental Figure 2, A–D). Both RIG-I and MDA5 interact with MAVS and activate the antiviral signaling pathway. Since our data reveal no induction of the activation of the MAVS in rmCIRP-treated macrophages, the RIG-I–MDA5–MAVS pathway may be less implicated in eCIRP-mediated type I IFNs expression, reflecting the primary involvement of the STING pathway for type I IFNs production.

In our study, we used the global STING-KO mice. Given the availability of only the global STING^–/–^ mice, regardless of various haplotypes of STING as evident in humans, we only studied eCIRP’s effect on the STING pathway using global STING^–/–^ mice. In the context of STING’s isotypes, a recent study revealed a potentially novel transcript isoform of STING, designated STING-β, that dominantly inhibits innate nucleic acid sensing (28). STING-β suppressed the induction of IFNs, IFN-stimulated genes, and other cytokines by various immunostimulatory agents, including CDNs, DNA, RNA, and viruses. STING-β sequesters cGAMP and other transducer molecules to inhibit innate nucleic acid sensing dominantly (28). Given the potential of STING haplotypes in immune response, future studies of eCIRP’s effects on various haplotype-specific STING-KO mice in HS will be of great importance.

We found decreased expression of IL-1β in the lungs of STING^–/–^ mice compared with WT mice after HS. We speculate 2 models to demonstrate why IL-1β production was less in STING-deficient HS mice than WT mice. One model depicts the STING-mediated activation of signal 1. The activated STING translocates to the Golgi compartments, where it interacts with TBK1 or IκB kinase (IKK). IκBα phosphorylation by IKK results in the translocation of NF-κB to the nucleus and the corresponding transcriptional expression of IL-1β (29). In the other model, the decreased expression of IL-1β could be due to the inhibition of inflammasome components, given that a noncanonical function of cGAMP in inflammasome priming and activation has been reported (29, 30). Although our current focus is beyond studying the role of eCIRP on absent in melanoma 2 (AIM2) inflammasome activation and IL-1β production, we assessed AIM2 expression in rmCIRP-treated macrophages, since AIM2 also serves as an intracellular DNA sensor. Indeed, we found no change in the AIM2 expression (Supplemental Figure 2, A and E), indicating that eCIRP-induced mitochondrial DNA fragmentation activating STING is independent of the AIM2 pathway. A recent study has shown the negative regulatory role of the AIM2 inflammasome on STING pathway activation (31). They found increased cGAMP generation, STING aggregation, TBK1 and IRF3 phosphorylation, and IFN-β transcription in AIM2 inflammasome–deficient APCs upon cytosolic DNA exposure. Given the adverse regulatory effects of AIM2 on STING and unaltered expression of AIM2 following rmCIRP’s stimulation, we predict that establishing the link between AIM2 and STING in rmCIRP-treated cells could be challenging. Nevertheless, when we inhibited AIM2 expression by its siRNA, we found decreased production of IL-1β by the rmCIRP-treated macrophages (Supplemental Figure 2, F–H). This indicates that eCIRP can induce IL-1β production through the AIM2 pathway. We previously reported that eCIRP could directly induce NLR family pyrin domain containing 3 (NLRP3) inflammation in lung endothelial cells to cause pyroptosis and IL-1β production. In contrast, we still do not know the inflammasome’s specificity for CIRP activation and release during inflammation, which could be an exciting area of future research.

In HS, circulating levels of eCIRP and type I IFNs were reported to be markedly elevated (6, 32). In our previous study, where we treated HS mice with anti-IFNAR1 antibody to block type I IFN recognition, we found beneficial outcomes in reducing inflammation, ALI, and survival, suggesting the detrimental role of type I IFN in HS (32). Conversely, type I IFNs were protective in acute viral infections but can have harmful roles in bacterial infections and autoimmune diseases (33, 34). Since the immune response to pathogen/DAMPs results in initial hyperinflammation followed by the late stage of immune suppression, both settings attribute to fatal complications. The type I IFN–mediated immune response to counteract infection may be of interest and needs attention while targeting these cytokines. Following inflammatory insults, TNF-α, IL-6, and IL-1β are abruptly increased early in HS induction; therefore, we assessed their levels in the early time point after HS. Mice were euthanized 4 hours after HS, so there was a lack of opportunity in this study to collect blood samples from these mice at later time points. In addition, there were several reasons we only measured cytokines at 4 hours after HS. We tried to minimize the number of animals by performing experiments at a single time point, as multiple sampling in the mouse after HS would adversely affect cardiovascular stability. We have performed 10-day survival studies, reflecting the outcomes of WT versus STING^–/–^ mice at the prolonged time period after HS induction. Nonetheless, the lack of cytokine data at time points later than 4 hours after HS is a limitation of our study.

Only male mice were used in this study. It has been reported that female sex steroids exhibit diverse immunomodulating functions in both humoral and cell-mediated immune responses under normal conditions, as well as various disease processes (35). Female sex hormones are protective in inflammation. Given the impact of female sex hormones on innate immune function, only male mice were used to generate reliable and consistent findings. However, in our future studies, mice from both sexes will be used. In our histology samples, we did not insufflate the lungs to a given pressure before fixation to provide uniformity, particularly when assessing septal thickening, which we confess to be the limitation of the histologic study.

In summary, these findings depict the detrimental role of STING in sterile ischemic injury. STING deficiency has protective effects in HS, as shown by decreasing organ damage and inhibiting proinflammatory cytokine and chemokine production in the lungs. HS causes the release of higher levels of CIRP in the blood. Macrophages sense the eCIRP via the TLR4-MyD88-TRIF pathway, leading to the activation of the STING pathway and type I IFNs. Finally, blockage of eCIRP-STING signaling could be a potential therapeutic target for mitigating HS-induced ALI.

## Methods

### Experimental animals.

Male C57BL/6 WT, STING^–/–^ (B6[Cg]-*Sting1^tm1.2Camb^*/J), TLR4^–/–^ ([B6(Cg)-*Tlr4^tm1.2Karp^*/J]), MyD88^–/–^ (B6.129P2-*Myd88^tm1Hlz^*/J), and TRIF^–/–^ (C57BL/6J-*Ticam1^Lps2^*/J) mice aged 8–10 weeks (20–25 g) were purchased from The Jackson Laboratory. The mice were housed under standard conditions (room temperature at 22°C, 12/12-hour light/dark cycle) with regular access to standard Purina mouse chow and water ad libitum. The animals were allowed at least 7 days to acclimate under these conditions before being used for experiments.

### Mouse model of HS.

HS in mice was induced by following our previous protocol (6, 32). In brief, mice were anesthetized with 2.5% inhaled isoflurane and then maintained with 1.5% inhaled isoflurane. The inguinal regions were then shaved and prepped with 10% povidone-iodine wash. Two 0.5 cm inguinal incisions were made to expose the femoral arteries from both sides, which were cannulated bilaterally with PE-10 (Becton Dickinson and Company) tubing after careful dissection of the femoral vein and femoral nerves. While 1 catheter was used for hemorrhage and resuscitation efforts, the second catheter was connected to a transducer to measure continuous MAP and heart rate. The results were recorded with a blood pressure analyzer Digi-Med (Micro-Med). The values of the MAP were averaged over 5-second intervals to record the oscillations in the blood pressure. Blood was withdrawn from the femoral artery to reach a MAP of 27.5 ± 2.5 mm Hg over 90 minutes. Mice were then resuscitated i.v. with Ringer’s lactate solution (Hospira) during the 30-minute resuscitation phase, with 2 times the amount of shed blood volume. Mice were not heparinized during the procedure. Each animal’s body temperature was kept at 37°C with a heating pad during the HS and resuscitation. After the resuscitation, the femoral artery was ligated and the incision was closed in layers with a nonabsorbable suture. Animals were observed to recover from anesthesia prior to their return to their cages. Sham mice underwent the same procedure without induction of hemorrhage or resuscitation.

### Survival study.

The survival rate was determined in 2 groups of mice, WT (*n* = 10) and STING^–/–^ (*n* = 10) mice with HS. After the HS procedure demonstrated above, the mice were returned to their cages for recovery and allowed food and water ad libitum throughout the 10 days. The survival rate was recorded over 10 days, and any surviving animals were euthanized humanely on day 10.

### I.v. injection of rmCIRP.

rmCIRP was prepared in house (6). Briefly, rmCIRP was expressed in *E.coli* and purified using Ni^2+^-NTA column (Novagen). The quality of the purified protein was assessed by Western blotting. The level of LPS in the purified protein was measured by a Limulus amebocyte lysate (LAL) assay (Cambrex). Mice were randomly assigned to receive 5 mg/kg body weight rmCIRP or equivalent volume PBS (vehicle). After induction of anesthesia, mice were placed supine, and the area over the right jugular vein was shaved and prepped with povidone-iodine Prep Pad (Professional Disposables International Inc.). An incision was made, and dissection was carried out to the level of the jugular vein. This was then isolated, and rmCIRP or PBS was injected. The vein was then ligated with nonabsorbable suture, and the incision was closed. Animals were observed to recover from anesthesia and were then returned to their cages. At 4 hours after injection, all mice were killed by CO_2_ asphyxiation, and organs were collected for processing.

### I.t. injection of rmCIRP.

WT mice were i.t. injected with rmCIRP (5 mg/kg body weight) or equivalent volume PBS as described previously. After induction of anesthesia by 2% isoflurane inhalation, mice were placed at the supine position, and the trachea was exposed to deliver rmCIRP or PBS using a 29G × 1/2” U-100 insulin syringe (Terumo Medical Corporation). The total volume of rmCIRP or PBS for i.t. administration was less than 50 μL. At 4 hours after injection of rmCIRP or PBS, all mice were killed by CO_2_ asphyxiation, and blood and lung tissues were collected for various analyses.

### Histology.

Before fixation, the lungs were harvested, where BAL was not performed, and perfusion was not done before histology in order to maintain its architectural integrity. Lung lobes from WT and STING^–/–^ mice were collected 4 hours after HS and stored in 10% formalin prior to fixation in paraffin. They were subsequently sectioned into 5 μM cuts and stained with H&E. Histologic lung injury was assessed in a blinded fashion using light microscopy evaluation, and severity of injury was scored using a system for the assessment of ALI in experimental animals outlined by the American Thoracic Society (36). The weighted score took into account neutrophil infiltration in the alveolar and interstitial spaces, the presence of hyaline membranes, the presence of proteinaceous debris in the airspaces, and the degree of septal thickening. Six fields/group were obtained from 6 mice/group, indicating 1 cross-section per mouse, at 200× magnification, with scores ranging between 0 and 1 for each sample.

### Analysis of tissue injury markers.

Blood samples were drawn prior to euthanasia from the inferior vena cava and centrifuged at 1000*g* for 10 minutes at 4°C. The supernatant containing the serum was collected and then analyzed immediately for levels of aspartate aminotransferase (AST), alanine aminotransferase (ALT), and lactate dehydrogenase (LDH) as organ injury markers using assay kits according to manufacturer’s instructions (Pointe Scientific).

### Measurement of cytokines by ELISA.

Whole blood was allowed to clot for 15–30 minutes after collection from the inferior vena cava. The serum was then separated by centrifugation at 1000*g* for 10 minutes at 4°C. The serum was collected and stored at –80°C until use. Serum levels of IL-6, IFN-β, and eCIRP were measured using ELISA kits specifically for these cytokines according to manufacturer instructions (IL-6, BD Biosciences, catalog 555240; IFN-β, Thermo Fisher Scientific, catalog 424001; CIRP, LifeSpan Biosciences, catalog LS-F16777). To measure proinflammatory cytokines in the lung, lung tissue was homogenized in lysis buffer (10 mM Tris-HCl [pH 7.5], 120 mM NaCl, 1% sodium deoxycholate, and 0.1% sodium dodecyl sulfate) (MilliporeSigma) containing a protease inhibitor cocktail (Roche Diagnostics). The protein concentration was then determined by the Bio-Rad protein assay reagent. Equal amounts of protein from lung homogenates were used to analyze the levels of TNF-α, IL-6, and IL-1β with a commercial mouse ELISA kit (BD Biosciences), according to the manufacturer protocol. Levels of IFN-α and IFN-β from the lungs and the supernatant of rmCIRP-treated peritoneal macrophages were measured using commercial mouse ELISA kits from Thermo Fisher Scientific (IFN-α, catalog BMS6027; IFN-β, catalog 424001) per the manufacturer’s instructions.

### Quantitative PCR (qPCR).

Whole lungs from WT and STING^–/–^ were harvested 4 hours after HS and flash-frozen at –80°C, and they were crushed over dry ice to a fine powder. Total RNA was extracted from crushed tissue using Trizol reagent (Invitrogen). Total RNA (2 μg) underwent reverse transcription using murine leukemia virus reverse transcriptase (Applied Biosystems). The qPCR reaction was carried out in a 20 μL final volume containing 0.25 μL each of forward and reverse primers, 2 μL cDNA, 7.5 μL DEPC-treated water, and 10 μL Power SYBR Green PCR Master Mix (Applied Biosystems). An Applied Biosystems StepOnePlus real-time PCR machine was used for amplification under the thermal cycling profile of 95°C for 10 minutes, followed by 40 cycles of 95°C for 15 seconds and 60°C for 1 minute. Mouse β-actin mRNA levels were used for normalization. Relative expression of mRNA was calculated by the 2^–ΔΔCt^ method, and results are expressed as fold change compared with the sham group. The primers used for qPCR are IFN-α, IFN-β, TNF-α, IL-6, IL-1β, CCL3, CXCL1, COX2, iNOS, and β-actin (Supplemental Table 1).

### Determination of myeloperoxidase activity.

WT and STING^–/–^ mice were subjected to sham or HS. Lung tissues were frozen in liquid nitrogen and stored at –80ºC, and MPO activity in the lungs was quantified. A total of 50 μg of lung tissue was homogenized in ice-cold PBS and centrifuged at 10,000*g* for 10 minutes in 4°C. The supernatant was discarded, and the pellet was resuspended and homogenized in potassium phosphate buffer (K_2_HPO_4_, 50 mM, pH 6.0) containing hexadecyltrimethylammonium bromide (HTAB, 0.5% w/v; MilliporeSigma). Following centrifugation at 3000*g* for 15 minutes at 4°C, the supernatant was collected, and MPO activity was quantified by measuring the H_2_O_2_-dependent oxidation of o-dianisidine. One unit (U) of MPO activity is defined as MPO levels per gram of lung tissue that caused a change in absorbance of 1.0/min at 450 nm.

### Western blotting.

Peritoneal macrophages treated with rmCIRP for 4 hours were homogenized in RIPA buffer (10 mM Tris-HCl [pH 7.5], 120 mM NaCl, 1% NP-40, 1% sodium deoxycholate, and 0.1% SDS; MilliporeSigma) containing a protease inhibitor cocktail (Roche Diagnostics) and using high-frequency sonication. Samples were then centrifuged at 10,000*g* for 15 minutes at 4°C, and the supernatant was collected for further analysis. Protein concentration was subsequently determined by DC protein assay (Bio-Rad). Equal amounts of tissue homogenates were fractionated on SDS-PAGE and transferred to nitrocellulose membrane. The membranes were blocked by incubation in 0.2× PBS with 0.1% casein and incubated overnight at 4°C with the following rabbit polyclonal antibodies: TBK1/NAD (Cell Signaling Technology; catalog 3504), pTBK1 (Cell Signaling Technology, catalog 5483S), IRF3 (Cell Signaling Technology, catalog 4302), pIRF3 (Cell Signaling Technology, catalog 4947), and mouse β-actin antibody (Sigma-Aldrich; catalog A5441) in 0.2× PBS with 0.1% casein and 0.1% Tween 20. After washing 3×, the blots were subsequently incubated with corresponding fluorescent secondary antibody (LI-COR). Bands were detected using the Odyssey FC Dual-Mode Imaging system 2800 (LI-COR).

### Isolation and stimulation of peritoneal macrophages.

Primary peritoneal macrophages were isolated from C57BL/6 WT, STING^–/–^, TLR4^–/–^, MyD88^–/–^, and TRIF^–/–^ mice day 4 after i.p. injection with 4% thioglycolate as previously described (37). Briefly, 1 mL of 3% thioglycolate (Sigma-Aldrich) was injected in mice i.p. Four days later, mice were euthanized using CO_2_ asphyxiation. Peritoneal fluids were isolated using peritoneal lavage with PBS. Total peritoneal cells were enriched by centrifugation at 300*g* for 10 minutes at 4°C and cultured in RPMI 1640 medium (Thermo Fisher Scientific). After 3 hours of culture, nonadherent cells were removed, and adherent cells, mainly the primarily macrophages, were cultured overnight. These primary peritoneal macrophages were treated with 5 μg/mL of rmCIRP for 4 hours. All cultured media was supplemented with 10% heat-inactivated FBS (MP Biomedicals), 1% penicillin-streptomycin (Thermo Fisher Scientific), and 2 mM glutamine (Thermo Fisher Scientific). Cells were maintained in a humidified incubator with 5% CO_2_ at 37°C.

### Immunofluorescence assay.

After rmCIRP (1 μg/mL), stimulation of peritoneal macrophages for 4 hours, the cell culture fluid was changed. MitoTracker Red (Invitrogen, catalog M7512) was added to the culture medium to a final concentration of 200 nM. After 1 hour of culture, the cells were fixed with 2% paraformaldehyde solution for 20 minutes and then washed with PBS and blocked with normal 5% BSA for 1 hour. The cells were stained with TUNEL following the manufacturer’s instructions (Roche Diagnostics). The cell nucleus was stained with Hoechst 33258 (Sigma-Aldrich). The macrophages were then measured by confocal microscopy (Olympus, Fluoview-FV1000). Cells were counted in 3 random fields in each independent experiment.

### Statistics.

We tested the data for normal distribution and performed analysis as they passed the normality test. Data represented in the figures are expressed as mean ± SEM. ANOVA was used for 1-way comparison among multiple groups, and the significance was determined by the Student–Newman–Keuls (SNK) test or the Tukey method, as appropriate. Two-tailed Student’s *t* test was applied for 2-group comparisons. Significance was considered for *P* ≤ 0.05 between study groups. Data analyses were carried out using GraphPad Prism graphing and statistical software (GraphPad Software).

### Study approval.

All experiments were performed by the guidelines for the care and use of experimental animals by the NIH. This protocol was approved by the IACUC of the Feinstein Institutes for Medical Research.

## Author contributions

KC, JC, and MA designed the experiments. JC performed HS in mice and conducted in vivo studies. KC, JC, and CT performed in vitro experiments. KC, JC, MA, and MB analyzed the data. KC, JC, MA, and MB prepared the figures. MA and KC wrote the manuscript. PW revised and edited the manuscript. MA and PW supervised the work. PW conceived the concept. The order of the co–first authors was determined based on their relative contribution in performing the experiments and drafting the manuscript.

## Supplementary Material

Supplemental data

## Version 1 07/22/2021

Electronic publication
