# Supplementary material for: Extracellular CIRP activates STING to exacerbate hemorrhagic shock
Source: JCI Insight. 2021 Jul 22;6(14):e143715. doi: 10.1172/jci.insight.143715 (PMC8410031; doi:10.1172/jci.insight.143715)
Supplement: Supplemental data [file jciinsight-6-143715-s113.pdf]

Supplemental Figure 1

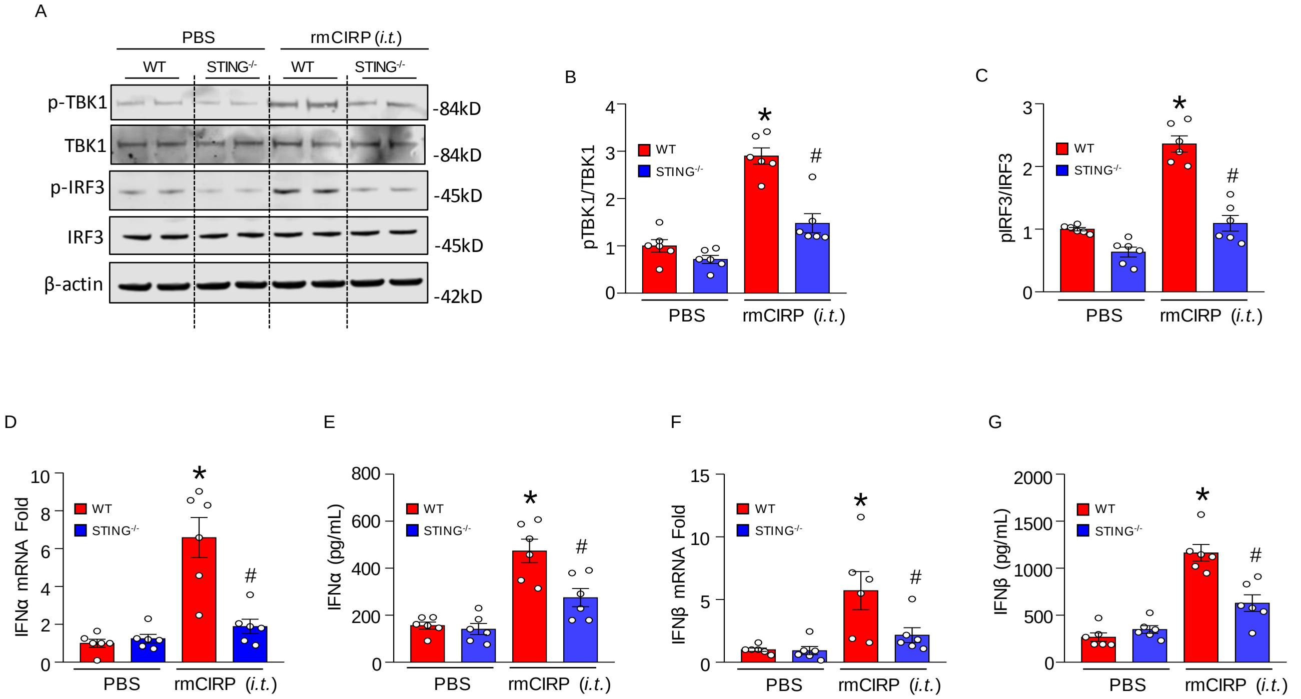

Supplemental Figure 1: Activation of TBK1 and IRF3 in lungs is decreased in STING$^{-/-}$ mice treated with rmCIRP intratracheally. (A-C) Assessment of pTBK1 and pIRF3 expression in lungs after intratracheal (*i.t.*) injection of WT and STING$^{-/-}$ mice with rmCIRP (5 mg/kg body weight). After 4 h of *i.t.* injection with rmCIRP or equivalent volume PBS (vehicle), lungs were harvested from each group of mice and assessed for **(A, B)** pTBK1 and TBK1 and **(A, C)** pIRF3 and IRF3 proteins by western blot. The blot was stripped and incubated with anti-β-actin Abs to serve as loading control. Representative western blots for pTBK1, TBK1, pIRF3, IRF3, and β-actin are shown. Each blot was quantified by densitometry analysis. pTBK1 and pIRF3 expression in each sample was normalized to total TBK1 and IRF3 expression and the mean values of WT PBS-treated group were standardized as one for comparison. **(D-G)** Lung tissues of PBS or rmCIRP-injected (*i.t.*) WT and STING$^{-/-}$ mice were analyzed for the expression of **(D, E)** IFNα, and **(F, G)** IFNβ at mRNA and protein levels by real-time PCR and ELISA, respectively. Data are expressed as mean ± SEM (*n* = 6

mice/group) and compared by ANOVA and SNK test (*p < 0.05 vs. PBS- and #p < 0.05 vs. rmCIRP-injected mice).

Supplemental Figure 2

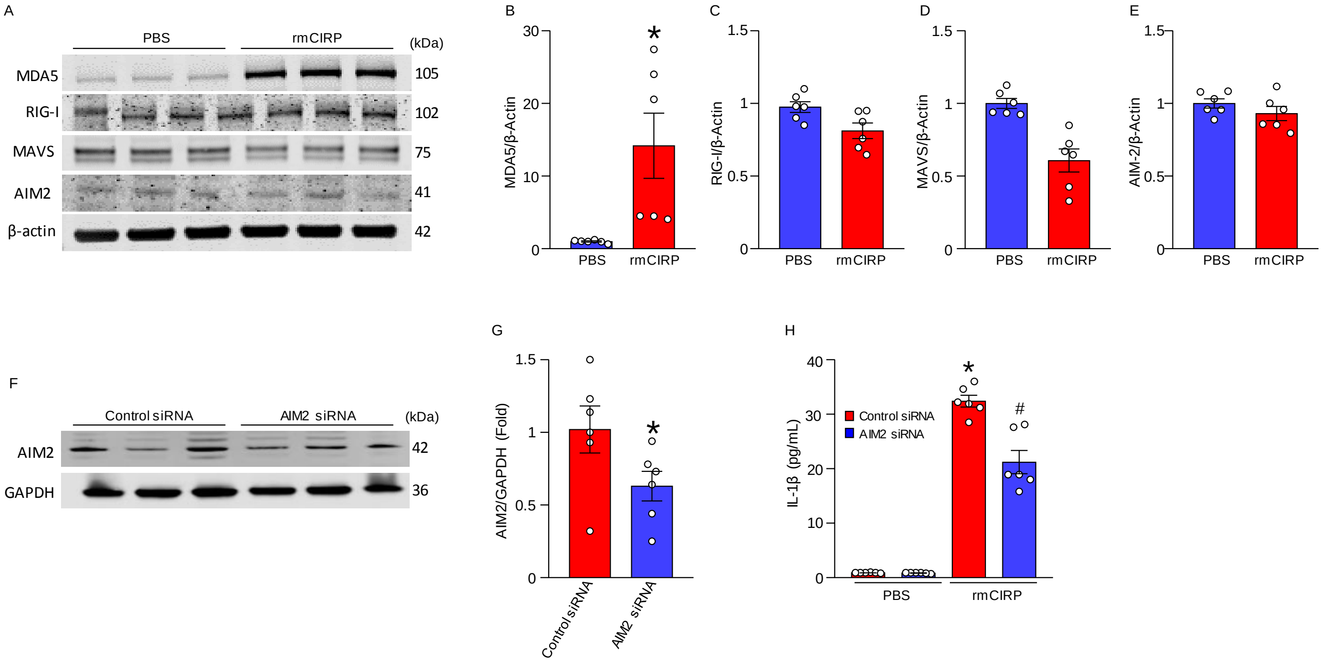

Supplemental Figure 2: MDA5, RIG-I, MAVS, and AIM2 expression in rmCIRP-treated macrophages and the effect of AIM2 inhibition on IL-1β production by rmCIRP-treated macrophages *in vitro.* **(A-E)** RAW264.7 cells (1×10^6 cells/mL) were stimulated with 1 μg/mL of rmCIRP or equal volume of PBS as vehicle control. After 4 h of stimulation with rmCIRP total protein was extracted from each sample and assessed for **(A, B)** MDA5, **(A, C)** RIG-I, **(A, D)** MAVS, and **(A, E)** AIM2 proteins by western blot. The blot was stripped and incubated with anti-β-actin Abs to serve as loading control. Representative western blots for MDA5, RIG-1, MAVS, AIM2, and β-actin are shown. Each blot was quantified by densitometry analysis. MDA5, RIG-1, MAVS, and AIM2 expression in each sample was normalized to β-actin expression, respectively and the mean values of PBS-treated group were standardized as one for comparison. Data are expressed as mean ± SEM (*n* = 6 samples/group) and compared by

Student's *t* test (*p < 0.05 vs. PBS-treated macrophages). The data passed the normality test (Shapiro-Wilk), and the subsequent two-tailed p value is <0.05. **(F, G)** Inhibition of AIM2 expression in J774A.1 cells by siRNA transfection. AIM2 siRNA (SR402231B) and negative control siRNA (SR30004) were purchased from OriGene Technologies (Rockville, MD). siRNA transfection reagent (sc-29528, Santa Cruz Biotechnology) was used for efficient transfection of AIM2 siRNA in to J774A.1 cells by following the manufacturer protocol. AIM2 siRNA: CCCAAAUAACGUUGUUAAGAGAGCC. After 24 h of transfection, cells were lysed and subjected to western blotting using anti-AIM2 Abs. The blot was stripped and incubated with anti-GAPDH Abs to serve as loading control. Representative western blots for AIM2, and β-actin are shown. Each blot was quantified by densitometry analysis and the results were expressed as fold change. Groups were compared by Student's *t* test (*p < 0.05 vs. NC-siRNA-treated macrophages). **(H)** J774A.1 cells (3×10^5 cells/mL) were seeded in to 12-well plates and transfected with control siRNA or AIM2 siRNA for 24 h. The siRNA-transfected cells were stimulated with rmCIRP (1 µg/ml) or PBS for 24 h. The level of IL-1β in the supernatants were detected by ELISA. Data are expressed as mean ± SEM (*n* = 6 samples/group) and compared by one way ANOVA and SNK test (*p < 0.05 vs. PBS-treated control siRNA-treated and $^{\#}$p < 0.05 vs. rmCIRP-treated control siRNA-treated macrophages).

Supplemental Table 1: qPCR primers.

**Name**	**Forward**	**Reverse**
IFNα	CTACTGGCCAACCTGCTCTC	AGACAGCCTTGCCAGGTCATT
IFNβ	TGACGGAGAAGATGCAGAAG	ACCCAGTGCTGGAGAAATTG
CCL8	ACATCACCTGCTTGGTCTGGAAAAC	ACTAAAGCTGAAGATCCCCCTTCG
CXCL2	CGCTGTCAATGCCTGAAGAC	ACACTCAAGCTCTGGATGTTCTTG
CXCL1	GCTGGGATTCACCTCAAGAA	ACAGGTGCCATCAGAGCAGT
TNFα	CTACTGGCCAACCTGCTCTC	AGACAGCCTTGCCAGGTCATT
IL-6	CCGGAGAGGAGACTTCACAG	GGAAATTGGGGTAGGAAGGA
CCL3	AGGTCCCTGTCATGCTTCTG	TCTGGACCCATTCCTTCTTG
COX2	CTCAGCCAGGCAGCAAATC	ACATTCCCCACGGTTTTGAC
iNOS	GCAGGTCGAGGACTATTTCTTTCA	GAGCACGCTGAGTACCTCATTG
β-actin	GTGAAAAGATGACCCAGATCA	TGGTACGACCAGAGGCATACAG
